# Treatment of mechanically-induced vasospasm of the carotid artery in a primate using intra-arterial verapamil: a technical case report

**DOI:** 10.1186/1471-2261-4-11

**Published:** 2004-07-21

**Authors:** Alexander L Coon, Geoffrey P Colby, William J Mack, Lei Feng, Philip Meyers, E Sander Connolly

**Affiliations:** 1 Department of Neurosurgery, Johns Hopkins Hospital, Baltimore, MD, USA; 2 Department of Neurological Surgery, Columbia University, New York, NY, USA; 3 Department of Radiology, Columbia University, New York, NY, USA

## Abstract

**Background:**

Despite improvements in the safety and efficacy of endovascular procedures, considerable morbidity may still be attributed to vasospasm. Vasospasm has proven amenable to pharmacological intervention such as nitrates, intravenous calcium channel blockers (CCBs), and intra-arterial papaverine, particularly in small vessels. However, few studies have focused on medium to large vessel spasm. Here we report the use of an intra-arterial CCB, verapamil, to treat flow-limiting mechanically-induced spasm of the common carotid artery (CCA) in a primate. We believe this to be the first such report of its kind.

**Case presentation:**

As part of a study assessing the placement feasibility and safety of a catheter capable of delivering intra-arterial cerebroprotective therapy, a female 16 kg baboon prophylaxed with intravenous nitroglycerin underwent transfemoral CCA catheterization with a metallic 6-Fr catheter without signs of acute spasm. The protocol dictated that the catheter remain in the CCA for 12 hours. Upon completion of the protocol, arteriography revealed a marked decrease in CCA size (mean cross-sectional area reduction = 31.6 ± 1.9%) localized along the catheter length. Intra-arterial verapamil (2 mg/2cc) was injected and arteriography was performed 10 minutes later. Image analysis at 6 points along the CCA revealed a 21.0 ± 1.7% mean increase in vessel diameter along the length of the catheter corresponding to a 46.7 ± 4.0% mean increase in cross-sectional area. Mean systemic blood pressure did not deviate more than 10 mm Hg during the procedure.

**Conclusions:**

Intraluminal CCBs like verapamil may constitute an effective endovascular treatment for mechanically-induced vasospasm in medium to large-sized vessels such as the CCA.

## Background

Rapid advancements in endovascular technology and techniques allow for treatment of an ever-increasing range of neurovascular diseases. Despite improvements in the safety and efficacy of these procedures, complications such as vasospasm, stroke, and perforation still occur [1]. Vasospasm, or contraction of smooth muscle fibers in the wall of a vessel, is a commonly recognized adverse event that may complicate an endovascular procedure by limiting distal blood flow.

Vasospasm complicates many disease states, particularly those affecting small vessels. Recently, treatment of small-vessel vasospasm has proven amenable to pharmacological intervention. For example, in the treatment of cerebral artery spasm, intravenous nitrates [2], intravenous calcium channel blockers (CCBs) [3], and intra-arterial papaverine [4] and CCBs [5] have been shown to prevent or mitigate this small artery spasm. However, few studies have focused on the treatment of medium and large vessel spasm [6], and even fewer have taken aim at mechanically-induced vasospasm. This type of spasm, unlike subarachnoid hemorrhage-induced vasospasm, is not the result of inflammation [7] and a functional nitric oxide deficiency [8,9], but rather direct physical irritation of the endothelium. In this report, we demonstrate the use of an intra-arterial CCB, verapamil, to treat flow-limiting mechanically-induced spasm of the common carotid artery (CCA) in a non-human primate. We believe this to be the first such report of its kind.

## Case presentation

As part of a study aiming to assess the placement feasibility and safety of a catheter capable of delivering intra-arterial cerebroprotective therapy, a 16 kg female baboon (*Papio anubis*) underwent carotid artery catheterization under general anesthesia. Since *Papio anubis *is regarded as vasospasm-prone (unpublished data), the animal was pre-treated with oral nimodipine (Nimotop, Bayer,1 mg/kg every 4 hours for 24 hours), and placed on a prophylactic infusion of intravenous nitroglycerin (200 mcg/hr) and heparin (100 units/hr). To place a 6 Fr (2 mm) treatment device in the 3–4 mm right CCA [10], the animal underwent transfemoral catheterization with a 7 Fr guiding catheter using Seldinger technique. Under single-plane fluoroscopic guidance, the guiding catheter was placed into the brachiocephalic artery (5–6 mm) and then advanced into the right CCA after prophylactic administration of 2 mg of intra-arterial verapamil (1 mg verapamil/cc normal saline). A proprietary 6 Fr metallic catheter was then passed through the guiding catheter. Once inside the CCA, the 6 Fr catheter was exposed by retraction of the 7 Fr guiding catheter. Control arteriography, performed by injection of non-ionic iodinated contrast material through the guiding catheter, revealed normal patency of the carotid artery without evidence of spasm or limitation of arterial flow. As part of the study protocol, this co-axial catheter system remained in the brachiocephalic vessels for 12 hours. Throughout this procedure, the animal was maintained under general anesthesia with a narcotic-nitrous mixture. Intravenous nitroglycerin infusion (200 mcg/hr) and physiological monitoring were continued. The guiding catheter was connected to a heparinized saline infusion (3 units heparin/cc normal saline at a rate of 30 cc/hour).

**Figure 1:**
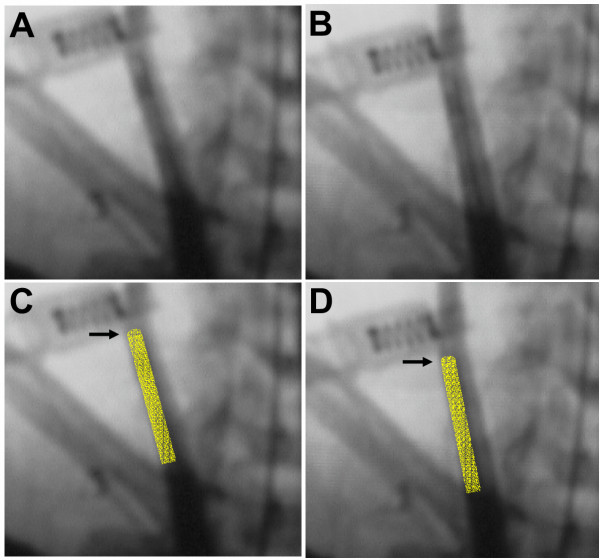
Anterior-posterior angiogram of right common carotid artery injection of a *Papio anubis *with a 6 Fr catheter in place both (**A.**) during vessel spasm on catheter, and (**B.**) 10 minutes after infusion of intraluminal verapamil (2 mg). Overlay images showing 6 Fr catheter position in CCA (gold) during spasm (**C.**) and after alleviation with verapamil (**D.**). Arrows (→) indicate tip of catheter.

**Figure 2:**
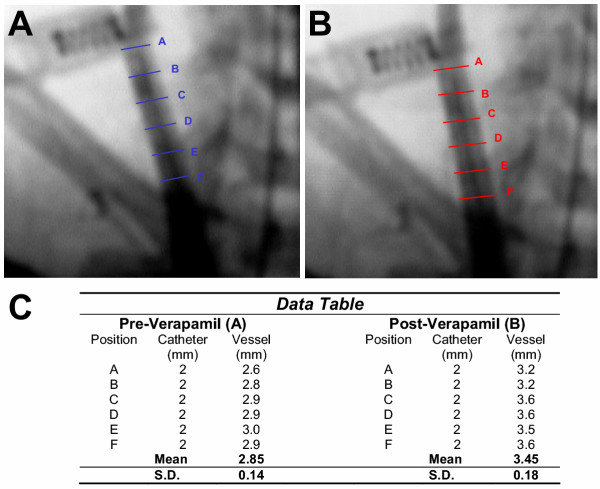
Image analysis at 6 paired positions (Lines A-F) along catheter in common carotid artery both (**A.**) during vessel spasm, and (**B.**) 10 minutes after intraluminal verapamil (2 mg) administration. (**C.**) Raw data table includes vessel diameter measurements both pre and post-verapamil injection.

**Figure 3:**
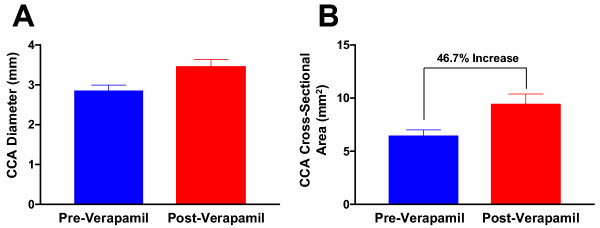
(**A.**) Bar graph depicting both the pre and post-verapamil mean vessel diameters from six positions along the length of the common carotid artery (CCA) (2.85 ± 0.14 mm and 3.45 ± 0.18 mm, respectively), and (**B.**) cross-sectional areas (6.41 ± 0.61 mm^2 ^and 9.39 ± 1.0 mm^2^, respectively). Note the 46.7% increase in mean cross-sectional area after verapamil administration.

Before removing the co-axial system at the conclusion of the experiment, carotid arteriography was performed to verify positioning of the catheter and patency of the vessels. These images revealed a decrease in vessel diameter localized to the length of artery where the 6 Fr catheter was positioned (Fig. 1A and 1C). Prior to further manipulation of the catheters, an additional bolus of intra-arterial verapamil (2 mg/ 2 cc normal saline) was instilled through the guiding catheter positioned in the brachiocephalic artery. After ten minutes, repeat carotid arteriography demonstrated a visible increase in vessel caliber, presumably due to a reduction in vasospasm (Fig. 1B and 1D). The diameter of the CCA was compared before and after verapamil administration at 6 equally-spaced points along the catheter. This revealed an increase in the mean CCA diameter from 2.85 ± 0.14 mm during spasm to 3.45 ± 0.18 mm post-verapamil administration (Figure 2). This corresponded to a 21.0 ± 1.7% mean increase in the vessel diameter post-verapamil injection, which represents a 46.7 ± 4.0% mean increase in cross-sectional area (Fig. 3). Review of continuous invasive blood pressure tracings demonstrated minimal systemic response to the intra-arterial administration of verapamil at this dosage; systemic blood pressure did not deviate more than 10 mm Hg systolic following instillation of verapamil.

At the conclusion of the procedure, the co-axial catheter system was removed. Anesthetics, heparin, and nitroglycerin infusions were discontinued. The animal was awakened from anesthesia uneventfully showing no signs of neurological impairment. MRI brain scan, including diffusion-weighted imaging at 36 hours, showed no evidence of cerebral infarction.

## Discussion

Driven by technology and the ever-increasing need for minimally invasive treatment modalities, the number of endovascular procedures performed annually continues to rise. The increased number and variety of endovascular procedures have introduced new situations in which vasospasm may be encountered. The sheer size and complexity of large bore catheters and their delivery systems makes them more likely to induce spasm in the vessel in which they are utilized (medium and large caliber arteries). Thus, it is important to identify pharmacological agents that will relieve this vasospasm with minimal side effects.

Spasm of arteries secondary to therapeutic medications or diagnostic instrumentation has long been acknowledged as a possible complication of interventional procedures. Vasospasm, in general, has been attributed to a variety of pharmacological stimuli ranging from cocaine [11] and alcohol [12], to L-thyroxine [13] and NSAIDs [14]. Vasospasm may also be attributed to mechanical irritation [15], as in the present study. In the past, treatment of mechanical spasm has simply been withdrawal of the offending catheter. A passive treatment such as this is often times undesirable, especially when the catheter system needs to remain in position, as in our experiment.

There are several agents that have been shown to be effective in preventing and treating vasospasm, each of which has its limitations. Intravenous nitrates have been the mainstay of vasospasm prevention for endovascular procedures [16], but their cardiovascular and intracranial pressure (ICP) effects limit their acute use for vasospasm treatment [17]. Intra-arterial papaverine has been used either as monotherapy or as an adjunct to balloon angioplasty in subarachnoid hemorrhage-induced vasospasm of smaller cerebral vessels [4,18,19]. However, papaverine therapy is short-acting, has untoward side-effects, such as elevation of ICP, and its role in larger vessel spasm remains ill-defined [20]. Recently, novel intra-arterial agents, such as mannitol and amrinone, have been used to reverse acute carotid spasm [21] and cerebral vasospasm following subarachnoid hemorrhage [22], respectively. Further efforts are needed to identify, compare, and validate pharamacotherapies for medium to large vessel spasm.

To reduce the tone of a muscular artery, a logical point of intervention is inhibition of calcium influx into smooth muscle cells. Voltage-sensitive CCBs, or 1, 4-dihydropyridines (such as verapamil, nifedipine, nimodipine, and amlodipine), function in this manner. Nimodipine improves outcomes after cerebral vasospasm secondary to subarachnoid hemorrhage [23]. Intraluminal administration of verapamil, in particular, has been used for both the pretreatment of vessels for endovascular procedures [16], as well as reversal of spasm in coronary grafts [6]. Recently, intra-arterial verapamil has also been reported to be safe and effective in the treatment of cerebral vasospasm [5]. Verapamil is well tolerated systemically, yet hypotension is the primary concern during administration.

In this case report we document the use of a 2 mg intra-arterial verapamil injection into the CCA of a non-human primate to acutely reverse catheter-induced vasospasm. This use is unique for several reasons. Firstly, in contrast to the report by He et al. [6], in which intra-arterial verapamil was used to alleviate spasm of an internal thoracic artery graft, we used verapamil to treat a significantly larger caliber vessel. Secondly, the presence of elastic fibers in the larger carotid artery compared to the highly muscular internal thoracic artery represents a different functional architecture. Thirdly, He et al. [6] attributed their observed vasospasm to ionotrope therapy (dobutamine, dopamine, and epinephrine) that was instigated for post-operative hemodynamic support. The spasm that we observed, however, occurred in the presence of hemodynamic stability and was isolated to a segment of the carotid in close association with a metallic 6 Fr catheter, suggesting mechanical irritation as the etiology.

This report serves as preliminary evidence for the utility of intra-arterial verapamil in large vessel vasospasm and not the conclusive study as its scope is limited by three issues. First, by not having a control (untreated) subject, it is impossible to say for sure that the observed mitigation of vasospasm is due to the intervention and not the natural history of the disease. Considering that effects were observed in the presence of an *in situ *metallic catheter, we believe strongly that the vasodilation was due to the verapamil. Second, the observed mitigation of vasospasm occurred during a continuous intravenous infusion of nitrates. It is conceivable that the synergistic effects of verapamil with these nitrates actually treated the vasospasm, and not the verapamil itself. Finally, this study, by its design, does not attempt to define the long-term durability of intra-arterial verapamil. Only through additional experimentation and use will the full utility of this agent as an intraluminal treatment for vasospasm be understood.

## Conclusions

We describe the acute alleviation of *in situ *catheter-induced CCA vasospasm in a non-human primate by an intra-arterial infusion of verapamil (2 mg) without demonstrable complications. Although only an observational study in one subject, this report suggests that intra-arterial administration of verapamil may be an effective intervention for the treatment of mechanically-induced vasospasm in medium to large-sized muscular arteries and that further experimentation in this area is warranted.

## Competing interests

None declared.

## Authors' contributions

ALC, GPC, and WJM performed the surgical procedure, delivered the critical care to the animal, composed, and revised the manuscript. LF and PM performed the angiography studies. ESC conceived the study and oversaw its design and completion.

## Pre-publication history

The pre-publication history for this paper can be accessed here:


